# A pancreatic mixed neuroendocrine-non-neuroendocrine neoplasms (MiNEN) (NET and undifferentiated carcinoma of the pancreas with osteoclast-like giant cells) with metastatic neuroendocrine component to the liver

**DOI:** 10.4322/acr.2020.201

**Published:** 2020-12-08

**Authors:** Guofeng Gao, Amir Qorbani, Chihong Heidi Zhou

**Affiliations:** 1 University of California, Davis Medical Center, Department of Pathology & Laboratory Medicine, Sacramento, CA, USA; 2 University of California, San Francisco Medical Center, Department of Pathology & Laboratory Medicine, San Francisco, CA, USA

**Keywords:** Pancreatic Neoplasms, Ductal Carcinoma of the Pancreas, Neuroendocrine Tumors, Carcinomas

## Abstract

Undifferentiated carcinoma of the pancreas with osteoclast-like giant cells (UCOGCs) is an extremely rare morphologically and clinically distinct variant of pancreatic ductal adenocarcinoma (PDAC), exhibiting a characteristic component of reactive osteoclast-like giant cells admixed with neoplastic mononuclear cells. Sommers and Meissner first described it in 1954 as an “unusual carcinoma of the pancreas”. Later it acquired many different names. In 2010, the WHO classified these tumors as a variant of PDAC under the heading of “undifferentiated carcinoma with osteoclast-like giant cells”. Here we describe the first case of pancreatic mixed neuroendocrine-non-neuroendocrine neoplasms (MiNEN) composed of UCOGC and pancreatic neuroendocrine tumor (NET), which occurred in a 78-year-old man with biliary colic and pancreatitis. The mass did not respond to the chemotherapy, and he soon developed liver metastasis from the NET component, and unfortunately, the patient passed away 10 months later. Since UCOGC is extremely rare, and its association with NET has not been reported yet, our case expands the knowledge regarding its unusual presentation and poor prognosis.

## INTRODUCTION

Pancreatic cancer is among the most devastating and lethal of all cancers. It comprises just 3 percent of all cancer cases in the United States, but its incidence is increasing. Some studies estimated the pancreatic cancer to become the second leading cause of cancer-related death by 2030.^1^ More than 55,000 Americans are estimated to be diagnosed with pancreatic cancer in 2018 (3.2% of all new cancers, 11^th^ most common cancer with a roughly equal gender predilection). However, due to the dismal 5-year survival rate (less than 9%), more than 44,000 will die from the disease in 2018 (7.3 percent of all cancer deaths), which makes it, currently, the third leading cause of cancer-related deaths in the United States.^2^ The vast majority of pancreatic cancers are pancreatic ductal adenocarcinoma (PDAC), an invasive mucin-producing gland-forming neoplasm with common somatic mutations involving KRAS oncogenes and *TP53*, *CDKN2A*, and *SMAD4* tumor suppressors.^3^

Undifferentiated carcinoma of the pancreas with osteoclast-like giant cells (UCOGCs) is an extremely rare variant of PDAC with an incidence of less than 1% of all pancreatic tumors.^4^ It was first described by Sommers and Meissner^5^ ^:101-111^ in 1954 as an 

“unusual carcinoma of the pancreas”. Later, it acquired many different names, like “carcinomas of pancreas simulating giant cell tumor of bone”, “osteoclastic giant cell tumor or carcinoma,” and “pleomorphic carcinoma of the pancreas, giant cell carcinoma”. In 2010, the World Health Organization classified these tumors as a variant of PDAC under the heading “ undifferentiated carcinoma with osteoclast-like giant cells.”^6^ These tumors have a characteristic morphology consisting of neoplastic mononuclear cells, some with significant cytologic atypia, admixed with reactive large multinucleated osteoclast-like giant cells. The nuclei of the osteoclast-like giant cells are uniform. Some of the osteoclast-like giant cells are actively phagocytic. Despite their unique morphologic features, they have strikingly similar mutations to PDACs.^7^ ^,^ ^8^ Tumors of UCOGCs are typically large (several centimeters) and may exhibit polypoid intraductal or intra-ampullary growth, or cystic degeneration. They may arise in mucinous cystic neoplasm or intraductal papillary mucinous neoplasm.^9^ ^,^ ^10^ The mean age of patients with UCOGCs is 62 years, with an equal male to female ratio, and mean survival rate of 12 months.^6^ ^,^ ^11^

UCOGC, is characterized by undifferentiated/anaplastic malignant cells, mixed with nonneoplastic osteoclast-like giant cells. Although the histopathological hallmark of UCOGCs is the characteristic large, bland, multinucleated osteoclast-like giant cells, these cells are not neoplastic. Morphologic, immunohistochemical, molecular, and ultrastructural studies have demonstrated that they represent benign histiocytic cells likely recruited by chemotactic substances of the tumoral cells. They show phagocytic capability (may contain neoplastic cells engulfed in the cytoplasm) and express histiocytic markers (CD68, vimentin, and common leukocyte antigen (CD45), but not keratin). They also have wild type p53 staining pattern on IHC. Mononuclear neoplastic cells usually do not show any line of differentiation on immunohistochemistry studies, but some show epithelial differentiation by expressing cytokeratin and diffuse positive for p53 (mutant p53 staining pattern). Ultrastructural findings of these mononuclear cells may reveal epithelial differentiation (desmosomes, microvilli, and zymogen-like granules). Tumors may exhibit extensive fibrosis, hemorrhage, and even osteoid formation, which may lead to a nondiagnostic sample; therefore, careful cytologic examination and histologic sampling are important.^6^ ^,^ ^12^

Cytological examinations of fine-needle aspiration are typically hypercellular, composed of highly characteristic multinucleated osteoclast-like giant cells intermingled with a noncohesive round to spindled cells and pleomorphic, hyperchromatic nuclei, and prominent nucleoli.^13^

It is being described that many tumors of undifferentiated carcinomas with osteoclast-like giant cells contain a separate glandular component or are associated with a preinvasive neoplasm, such as a mucinous cystic neoplasm.^6^ ^,^ ^7^ ^,^ ^9^ ^,^ ^10^ However, up to this date, no possible association with neuroendocrine neoplasm (NET or NEC) component has been reported in patients with UCOCG. A review of the English-speaking literature was performed by the authors, using the PubMed database by searching the terms “undifferentiated carcinoma with osteoclastic giant cells of the pancreas”, and none of the reported UCOCGs was found to have a NET component.

Pancreatic endocrine neoplasms (PENs) are rare and account for approximately 2% of all pancreatic neoplasms. They are epithelial tumors with endocrine differentiation, commonly affect adults between 30 to 60 years of age with no sex predilection.^6^ Microscopically, these tumors are composed of uniform cuboidal cells with centrally located nuclei and finely granular cytoplasm arranged in nests or tubules. They can be functional (gastrinomas, glucagonomas, etc.) or nonfunctional. They may be present sporadically or related to a familial syndrome (such as multiple endocrine neoplasia, also known as MEN syndromes). Immunohistochemically, Synaptophysin, and Chromogranin A may be useful in confirming the diagnosis. They also can express CD56, PAX8, and CK7. The molecular genetics of Pancreas NETs differ significantly from those of pancreatic ductal adenocarcinoma. Pancreas NETs do not typically have mutations in *KRAS*, *CDKN2A*, or *SMAD4*, and only a small minority harbor *TP53* mutations.^14^ One of the most challenging aspects of these tumors is the prediction of their biologic behavior. The mitotic count and Ki-67 proliferation index seems to be the most important factor that correlates with prognosis. The 2019 WHO classification of neuroendocrine neoplasms of the gastrointestinal tract and hepatobiliary organs classifies the pancreatic neuroendocrine neoplasms into G1 NET (low grade well-differentiated, mitoses <2/10HPF and Ki67 index <3%); G2 NET (intermediate grade well-differentiated, mitoses 2–20/10/HPF or Ki67 index 3%–20%); G3 NET (high grade well-differentiated, mitoses >20/10HPF or Ki67 index >20%); neuroendocrine carcinoma (poorly-differentiated, mitoses >20/10HPF or Ki67 index >20%, including small cell type and large-cell type); and MiENE (mixed neuroendocrine-nonneuroendocrine neoplasm).^6^

Pancreatic MiNENs are neoplasms composed of morphologically recognizable neuroendocrine and non-neuroendocrine components. Mixed ductal-neuroendocrine carcinomas account for about 0.5-2% of all ductal adenocarcinoma and usually metastasize to lymph nodes and liver.^6^ MiNENs include a range of specific diagnostic entities in the pancreas. Here we report the first case of pancreatic MiNEN of undifferentiated pancreatic carcinoma with osteoclast-like giant cells and neuroendocrine tumor with metastatic neuroendocrine component to the Liver.

### Case Report

**Figure 1:**
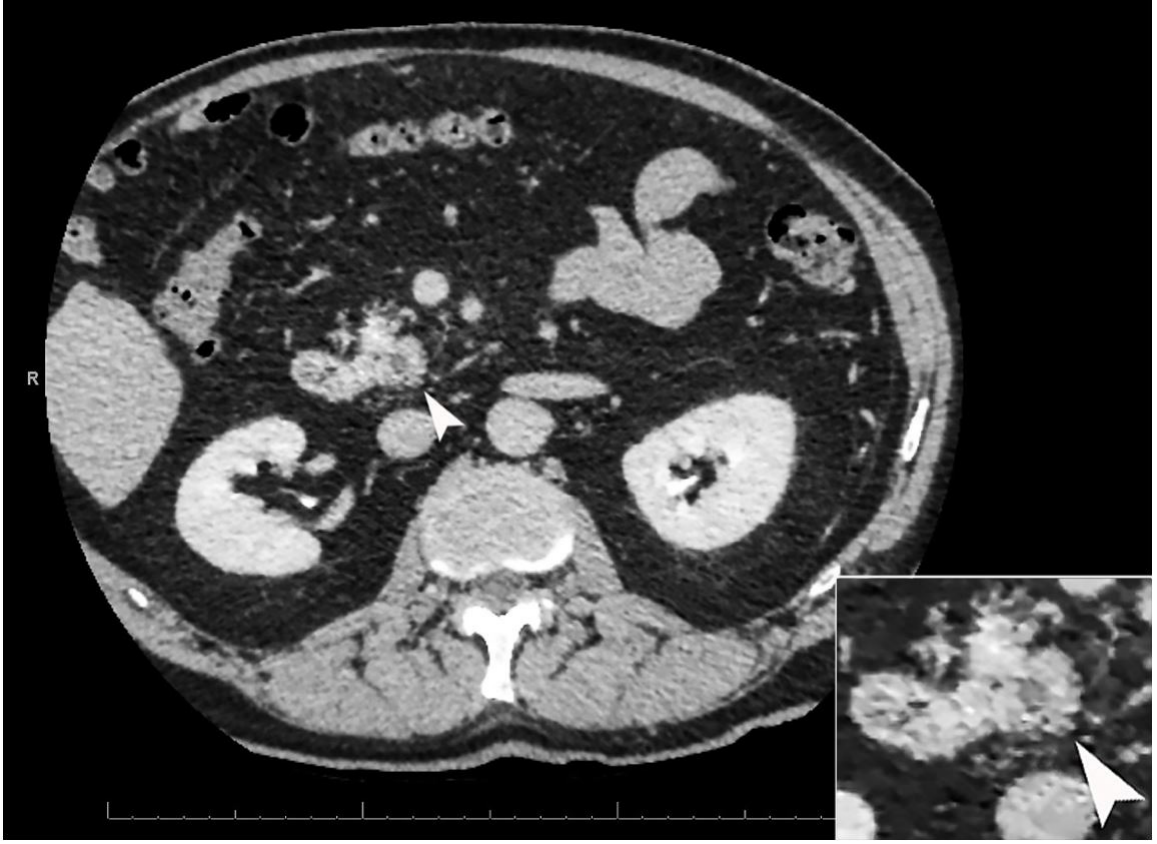
Abdominal axial computed tomography (CT) scan shows a 0.7 cm hypodense mass in the pancreas (arrow).

A 78-year-old man with a history of biliary colic presented with abdominal pain. The abdominal axial computed tomography (CT) scan showed a 0.7 cm hypodense lesion in the pancreas (Figure 1).

Endoscopic ultrasound (EUS) demonstrated a mass in the uncinate process of the pancreas. EUS-guided transduodenal fine needle aspiration (FNA) of the mass was performed.

**Figure 2:**
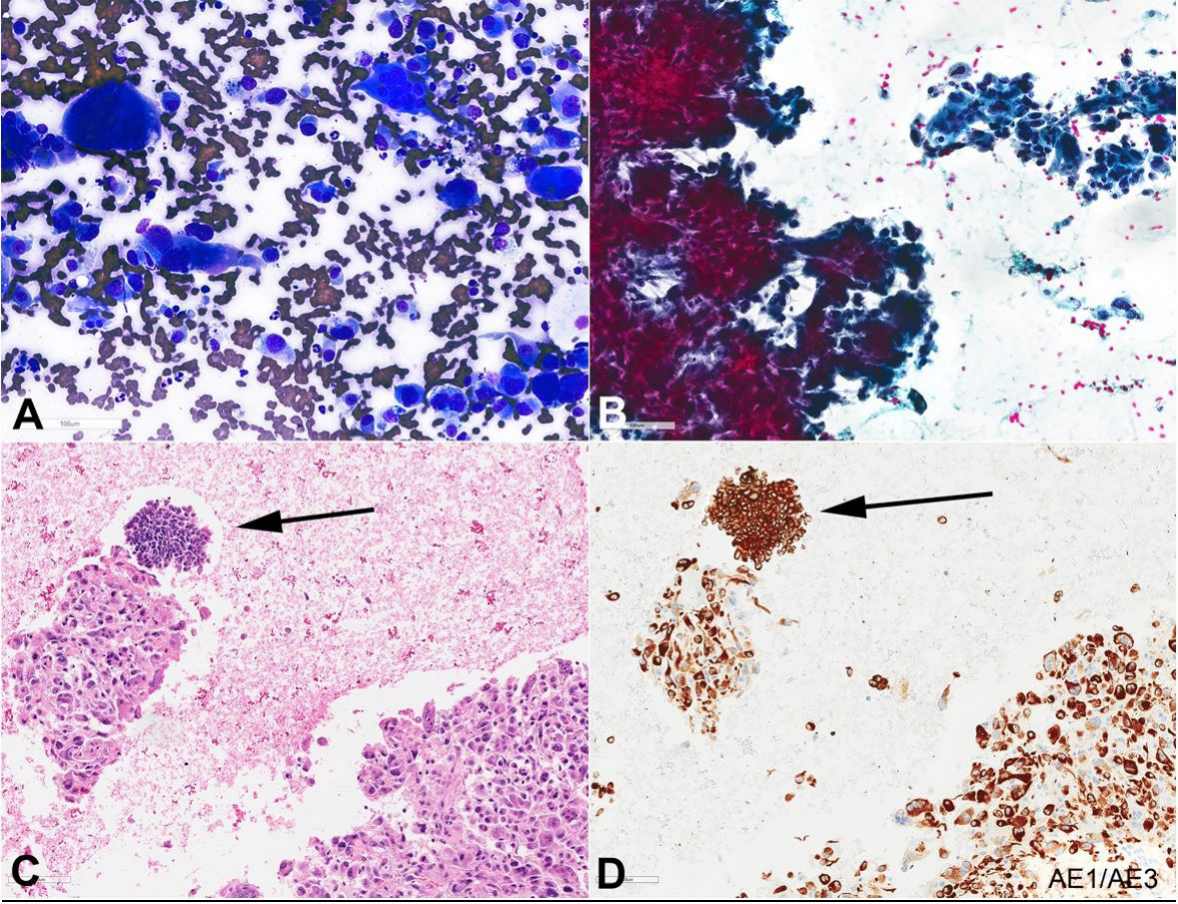
Photomicrographs of endoscopic ultrasound (EUS)-guided fine-needle aspiration biopsy (FNA) of the pancreas mass (400 x magnification); **A –** Diff-quick-stained smear showing hypercellular smear consists of pleomorphic tumor cells and osteoclast-like giant cells; **B –** Papanicolaou-stained smear showing hypercellular smear consists of cohesive spindle to pleomorphic tumor cells and osteoclast-like giant cells; **C –** H&E section of the cell block show pleomorphic and spindle cell neoplasm with scattered osteoclast giant cells (UCOGC component). Foci of smaller neoplastic round to spindle cells with scant finely granular cytoplasm and “salt and pepper” (finely stippled) chromatin are noted (neuroendocrine component; arrow); **D –** AE1/AE3 immunostain.

Smears by Diff-Quik stain and Papanicolaou stain were hypercellular and showed large tumor cells as crowded clusters, syncytial groups and singly dispersed, which were composed of three different types of cells: pleomorphic hyperchromatic tumor cells with irregular nuclear contours; multinuclear osteoclastic giant cells with smooth nuclear membranes, pale chromatin, and moderate cytoplasm; and some spindle-shaped tumor cells with slightly enlarged round to oval nuclei, irregular nuclear membranes, and moderate cytoplasm (Figures 22B). H&E sections from the cell bock showed similar morphologic features (Figure 2C). The immunohistochemistry study on the cellblock showed three different components (Table 1). The majority of tumor cells were diffusely and strongly positive for CK AE1/AE3, and p53, but negative for CD68, SMAD4, CK20, Glypican-3, CD56, Synaptophysin, TTF1, CA19-9, and HepPar1. Scattered osteoclast giant cells were noted, which were negative for epithelial markers (AE1/AE3, p53) but positive for CD68. A minor component of coexisting neuroendocrine neoplasm (<5%) was also noted, supported by different cytomorphology and different immunohistochemical stain pattern (small tumor cells positive for CK7, synaptophysin, CK19, CA19-9, SMAD4, CK AE1/AE3, and CD56; negative for Glypican-3, HEP PAR1, CK20, CD68, and PSA) (Figures 2 D, 33D, and 44D). The paucity of these neuroendocrine cells precluded the evaluation proliferation index by Ki67 immunostaining (it was observed as less than 20%).

**Table 1:** Histological and immunohistochemical patterns on the cell block

Component	AE1/AE3	P53	CD68	CK7	CK19	Syn	SMAD4	CD56	CK20	CA19-9
Pleomorphic cells	+	+	-	+/-	+/-	-	-	-	-	-
Osteoclast-like giant cells	-	-	+	-	-	-	-	-	-	-
Neuroendocrine cells	+	Weak/-	-	+	+	+	+	+	-	+

**Figure 3 gf03:**
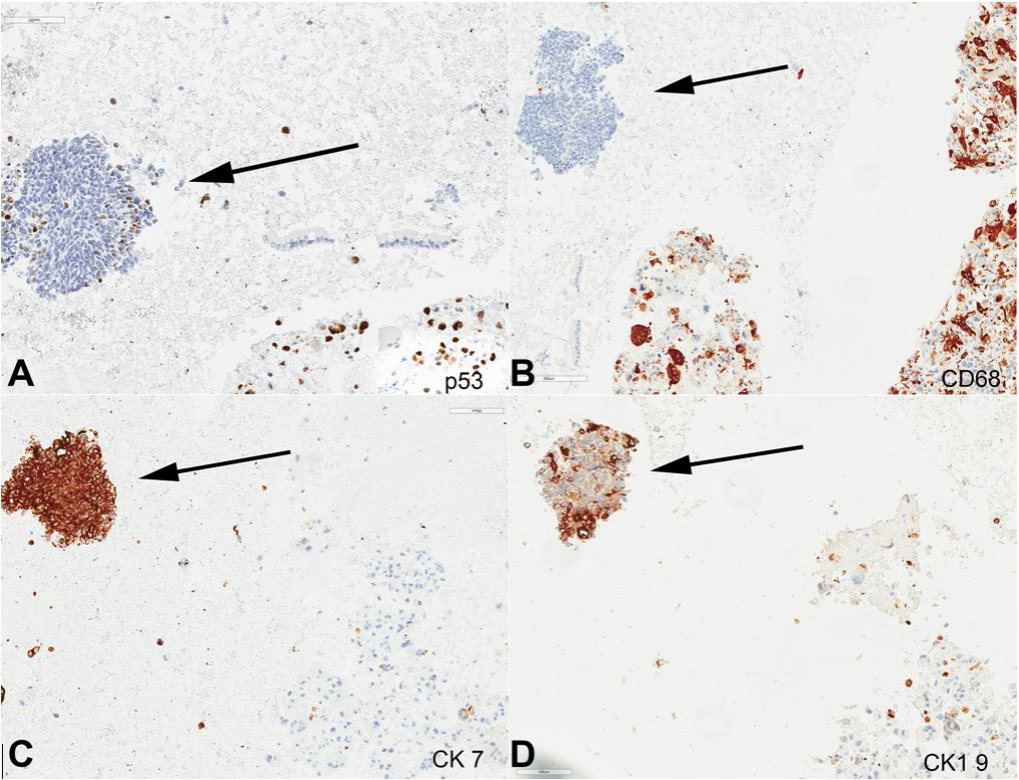
Photomicrographs of endoscopic ultrasound (EUS)-guided fine-needle aspiration biopsy (FNA) of the pancreas mass (400 x magnification), arrow indicates neuroendocrine component and the remaining cells are UCOGC component; **A –** P53 immunostain; **B –** CD68 immunostain; **C –** CK7 immunostain; **D –** CK19 immunostain.

**Figure 4:**
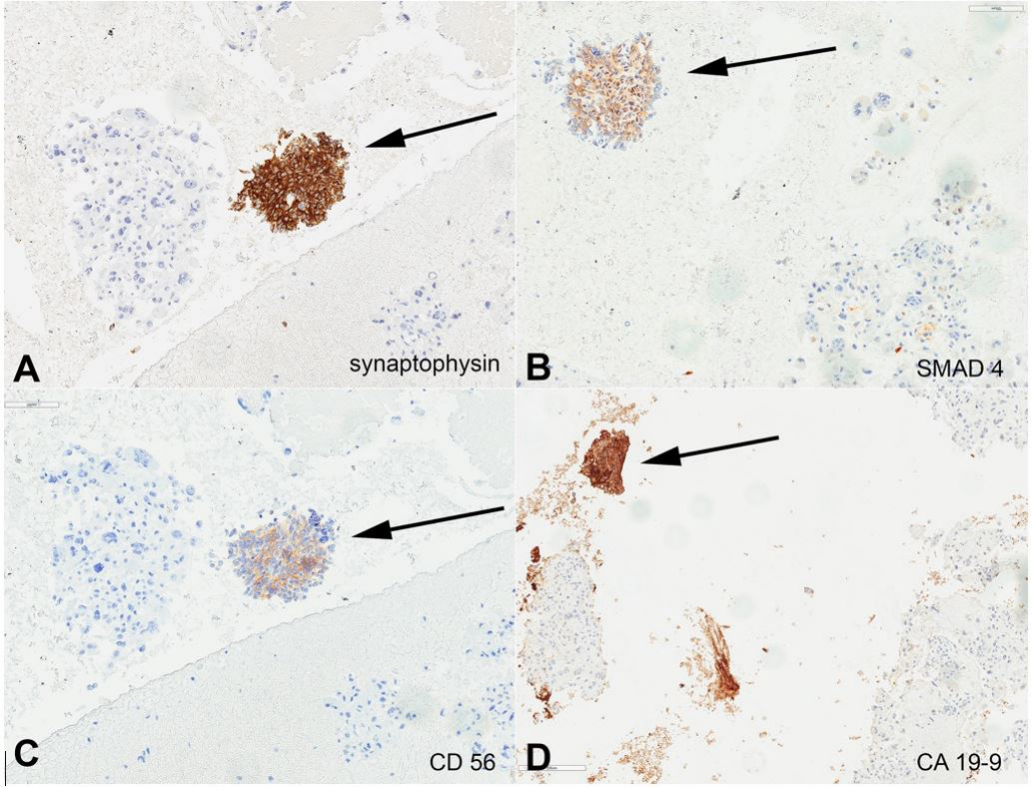
Photomicrographs of endoscopic ultrasound (EUS)-guided fine-needle aspiration biopsy (FNA) of the pancreas mass (400 x magnification) arrow indicates neuroendocrine component and the remaining cells are UCOGC component; **A –** Synaptophysin immunostain; **B –** SMAD4 immunostain; **C –** CD56 immunostain; **D –** CA19-9 immunostain.

**Figure 5:**
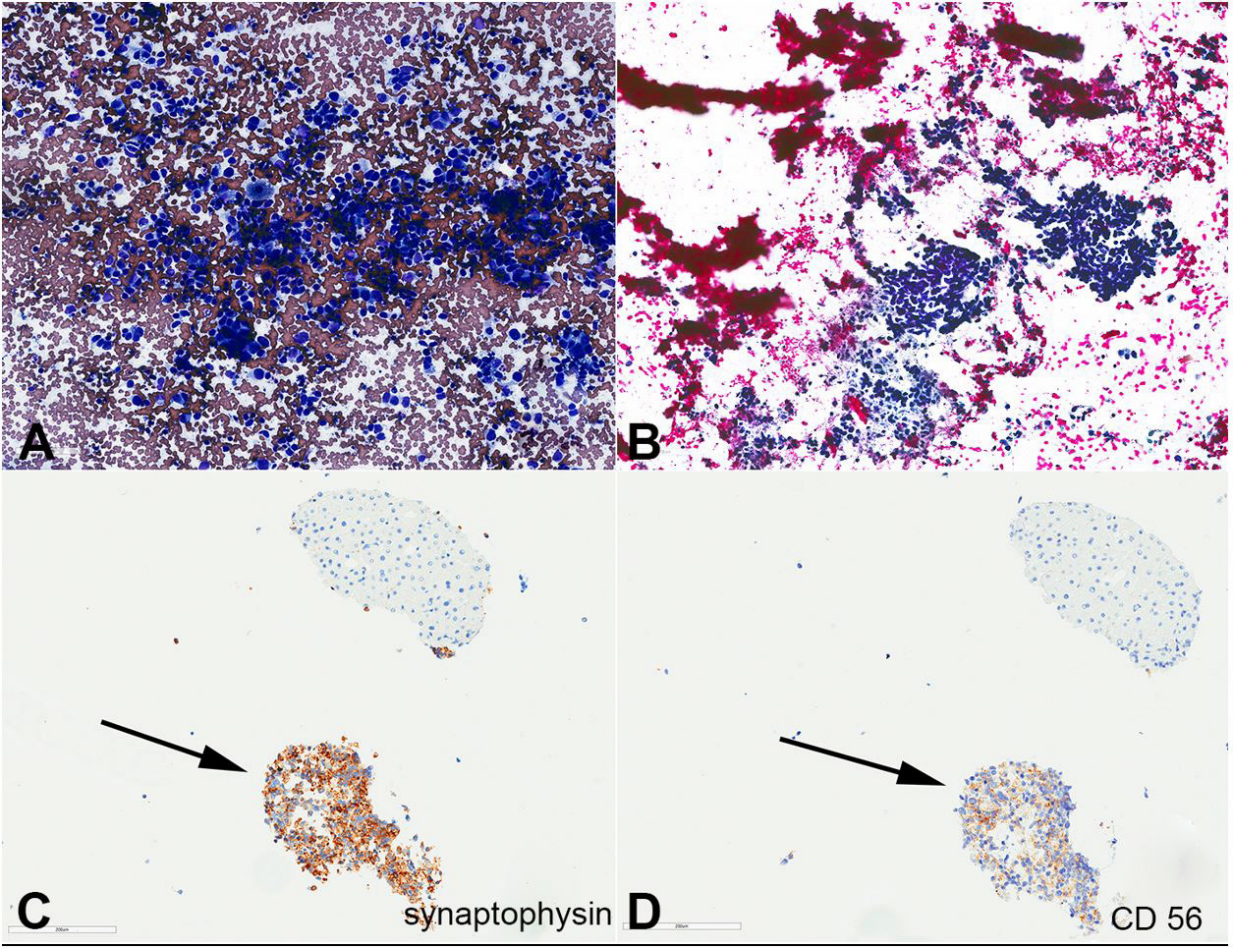
Photomicrography of endoscopic ultrasound (EUS)-guided fine-needle aspiration biopsy (FNA) of liver mass (400 x magnification), arrow indicates neuroendocrine component and the remaining cells are UCOGC component; **A –** Diff-quick-stained smear showing hypercellular smear consists of pleomorphic tumor cells with round to spindle nuclei, finely stippled chromatin, and scant cytoplasm; **B –** Papanicolaou-stained smear; **C –** Synaptophysin immunostain on the cell block show positive staining in neoplastic cells and negative staining in hepatic cells; **D –** CD56 immunostain on the cell block show positive staining in neoplastic cells and negative staining in hepatic cells.

While the patient was waiting for chemotherapy, an abdominal magnetic resonance imaging (MRI) showed a marked increase in the size of pancreatic mass with multiple small enhancing liver lesions. EUS-guided FNA of the liver mass revealed a neuroendocrine tumor composed of cells with similar cytomorphological features to the above neuroendocrine component of UCOGCs, supported by Immunohistochemical stains (positive for synaptophysin, CD56, and CK AE1/AE3; negative for HEP PAR1, Glypican-3, and CD45) (Figures 5AD). Ki-67 proliferation index was approximately 30-40%. The patient initially received chemotherapy (paclitaxel plus gemcitabine combination), which he tolerated poorly. Therefore, paclitaxel was discontinued and he was resumed chemotherapy with gemcitabine alone. Unfortunately, he passed away 10 months after his initial diagnosis of UCOGCs.

## DISCUSSION

Pancreatic mixed neuroendocrine-nonneuroendocrine neoplasm (MiNEN) includes a range of specific diagnostic entities in the pancreas. Here we report the first case of pancreatic MiNEN of undifferentiated pancreatic carcinoma with osteoclast-like giant cells and neuroendocrine tumor with metastatic neuroendocrine component to the Liver.

Pancreatic undifferentiated carcinomas with osteoclast-like giant cells are currently considered as a distinct and rare variant of pancreatic ductal adenocarcinoma (PDAC), which was supported by their shared strikingly similar genetic alterations known to drive carcinogenesis, including activating mutations in the oncogene *KRAS* and inactivating mutations in the tumor suppressor genes *CDKN2A*, *TP53*, and *SMAD4*.^7^ The study of a series of 22 UCOGCs by Luchini et al.^7^ showed that only one non‐synonymous missense mutation was identified in SMAD4 in one of eight UCOGCs with whole exome sequencing (WES) analysis, and approximately half (9) of 19 UCOGCs had loss of *SMAD4* expression on formalin-fixed paraphing-embedded (FFPE) sections by immunohistochemistry, including the case with somatic mutation identified by WES and two other cases with likely homozygous deletions. Five other UCOGCs analyzed by WES were demonstrated as *SMAD4* wild‐type and had intact expression. A study by Tang et al.^15^ demonstrated that abnormal *p53*, *Rb*, or *SMAD4* expression in 11/33 cases of poorly differentiated NECs. Waddell et al.^16^ showed that PDAC almost all have *KRAS* mutations, and Hijioka et al.^17^ demonstrated that 6 of their 7 cases of poorly differentiated NECs (86%) had *KRAS* mutations. These studies suggest that pNEC and PDAC have genetic similarities and may share a common origin.^18^ These suggest that somatic mutations may not be the determinants of the unique phenotype of PDACs, UCOGCs, or NECs. However, it has been demonstrated that pancreatic NECs often show abnormal expression of *p53* and *RB1*, but retain expression of *DAXX* and *ATRX*, while pancreatic G3 NETs almost always have intact *TP53* (only occasionally mutated in G3 PanNET with metastatic progression) and *RB1*, and lose *DAXX* and *ATRX* in approximately half cases, similar to low grade (G1-G2) pancreatic NET,^15^ suggesting that pancreatic NECs do not derive from pancreatic NET.

In general, undifferentiated carcinomas follow a more aggressive behavior, but some studies showed that undifferentiated pancreatic carcinoma with osteoclast-like giant cells (UCOCGs) seems to predict improved prognosis. These findings, along with the presence of reactive OCGs in these tumors, raises the possibility that these OCGs are the result of the immune response that eliminates the PDAC component and results in improved prognosis. This hypothesis is further supported by observing the better prognosis in patients with UCOGC without *PD-L1* expression.^8^ Muraki et al.^19^ reviewed 38 cases of UCOCGs and showed that its survival rate is significantly better than that of PDAC (UCOCGs estimated 5-year survival rate of 59.1%). They also observed that 76% of UCOCGs had an invasive ductal component (PDACs), and 21% arose in an intraepithelial neoplasm (IPMN or MCN). However, to our best knowledge, the presence of a neuroendocrine neoplasm component (NET or NEC) has not been reported in patients with UCOCGs. Our patient is the first case of MiNEN composed of UCOCGs and NET component without having any observed ductal differentiation and with metastasis to the liver of the NET component.^6^ Despite a possible better prognosis suggested by the study by Muraki et al.^19^ for patients of UCOCGs without ductal differentiation, according to WHO, resectability of the carcinoma is the most important prognostic factor for MiNEN and patients rarely survive > 3 years. The 2-year and 5-year survival rates are 25% and 0%, respectively, although a study by Basturk et al.^20^ suggested mixed ductal-neuroendocrine carcinomas have a slightly longer median survival time than pure NECs. Unfortunately, despite chemotherapy, our patient died within 10 months of initial diagnosis, due to the aggressive clinical course of the neuroendocrine tumor component.

Pancreatic MiNENs include a range of specific diagnostic entities in the pancreas and is composed of morphologically recognizable neuroendocrine and non-neuroendocrine components. The neuroendocrine component can be neuroendocrine tumor or neuroendocrine carcinoma.^6^ In this case report, we reported the first pancreatic MiNEN with UCOCGs and neuroendocrine components with metastatic neuroendocrine to the liver; the two components demonstrated different histology and immunostain profiles (Table 1).

The UCOCGs tumor cells were diffusely and strongly positive for CK AE1/AE3, p53 (diffuse strong), and lost expression of *SMAD4*; but negative for CK20, Glypican-3, CD56, Synaptophysin, TTF1, CA19-9, and HepPar1. The minor component of neuroendocrine tumor cells (<5%) were positive for CK7, synaptophysin, CK19, CA19-9, SMAD4, CK AE1/AE3, and CD56; but negative for Glypican-3, HEP PAR1, CK20, CD68, and PSA) (Figures 2 [Fig gf03]-4). The neuroendocrine tumor cells were scarce and showed a Ki-67 proliferation index < 20%. Primary G3 PanNETs retain a well-differentiated histology, with characteristically “organoid” arrangements of the tumor cells (relatively uniform, round to oval nuclei, coarsely stippled chromatin, and finely granular cytoplasm) with solid/nesting, trabecular, gyriform, or sometimes glandular patterns. They have a Ki-67 proliferation index > 20% and usually < 55%; however, no upper limit has been defined for their mitotic index or the Ki-67 proliferation index. They almost always have intact *TP53* and *RB1*, and only occasionally, a G3 PanNET can develop a *TP53* mutation during its metastatic progression. PanNET may contain low-grade components (G1, G2), or may present as metastases. Uniquely, the neuroendocrine component of our reported case of MiNEN (NET and UCOGCs) showed metastasis of the neuroendocrine tumor component to the liver. On the other hand, pancreatic NECs have poorly differentiated histology (either small cell or large cell type), tumor cells of irregular hyperchromatic nuclei growing in sheets, or diffuse architecture, often with large confluent areas of necrosis. The small cell type NECs consist of small- to medium-sized, round to oval cells with scant cytoplasm and hyperchromatic nuclei with indistinct nucleoli. The large-cell type NEC is composed of large cells with vesicular nuclei showing prominent nucleoli and abundant eosinophilic cytoplasm. However, histologically, morphological features may not always be reliable in separating a well-differentiated NET from a poorly-differentiated NEC. Furthermore, there is no Ki-67 index cutoff to separate G3 NET and NEC (both > 20%), although an extremely high Ki-67 proliferation index (>75%) or a mitotic rate > 20 per 10 HPFs usually indicates a NEC. Pancreatic NECs often show abnormal expression of *p53* and *RB1*, but retain expression of *DAXX* and *ATRX*, which is lost in approximately half of pancreatic G3 NET cases, similar to low grade (G1-G2) pancreatic NET.^15^ According to the WHO, the histological classification of collision tumors include at least two different malignant components located in the same organ or anatomic site, without mixed or transitional area.^6^ Pancreatic collision tumors composed of both a neuroendocrine tumor and a ductal adenocarcinoma has been reported.^21^ ^,^ ^22^ However, to our knowledge, there is no reported case of a collision tumor of UCOGC with pancreatic NEN. Because we do not have a surgical biopsy or resection specimen to determine the transitional area or stroma between the two components, a collision tumor of UCOGC with pancreatic NEN cannot be ruled out. A diagnosis of UCOGC with neuroendocrine differentiation is not likely due to the different histological features and immunohistochemical profiles of the two components.

## CONCLUSION

Pancreatic undifferentiated carcinomas with osteoclast-like giant cells are a distinct entity. An extensive histological sampling of UCOGCs is important to exclude associated PDAC or high-grade neuroendocrine carcinoma, which significantly impacts prognosis. Our reported case was the first reported incidence of a pancreatic MiNENs of undifferentiated pancreatic carcinoma with osteoclast-like giant cells and neuroendocrine tumor with metastatic neuroendocrine component to the liver, which expanded our knowledge regarding the clinical prognosis of this rare entity.
